# Use of Conventional and Innovative Technologies for the Production of Food Grade Hop Extracts: Focus on Bioactive Compounds and Antioxidant Activity

**DOI:** 10.3390/plants11010041

**Published:** 2021-12-23

**Authors:** Veronica Santarelli, Lilia Neri, Katya Carbone, Valentina Macchioni, Paola Pittia

**Affiliations:** 1 Faculty of Bioscience and Technologies for Food, Agriculture and Environment, University of Teramo, Via Renato Balzarini 1, 64100 Teramo, Italy; vsantarelli@unite.it (V.S.); ppittia@unite.it (P.P.); 2 CREA Research Centre for Olive, Fruit and Citrus Crops, Via di Fioranello 52, 00134 Rome, Italy; katya.carbone@crea.gov.it (K.C.); valentina.macchioni14@gmail.com (V.M.)

**Keywords:** antioxidant activity, food-grade extract, high hydrostatic pressure, hops, polyphenols, ultrasounds

## Abstract

This study investigated the use of conventional and innovative extraction methods to produce food-grade hop extracts with high antioxidant capacity and content in bioactive compounds. Conventional extractions (CONV) were performed under dynamic maceration at 25 and 60 °C; innovative extractions were performed using two ultrasound systems, a laboratory bath (US) and a high-power ultrasound bath (HPUS), and a high-pressure industrial process. For CONV, US, and HPUS extractions the effect of the extraction time was also tested. Experimental results showed that extraction method, temperature, and time affect to a different extent the phenolic profile and have a significant effect (*p* < 0.05) on the total phenolic content, total flavonoid content, antiradical capacity (ABTS), chlorophyll *α*, and total carotenoids content. Overall, US and CONV 60 °C extractions showed the highest extraction efficiency for almost all the investigated compounds, however, the extraction method and time to be used strongly depends on the target compounds to extract.

## 1. Introduction

In food productions, the use of plant extracts represents a strategy of growing interest to replace the use of chemical and synthetic additives or ingredients with functional properties and technological functionalities and to satisfy the demand of the stakeholders and consumers for innovative, high quality, healthy, clean labels, and sustainable food products. Indeed, fruit and vegetable extracts, depending on their composition and content in secondary metabolites (e.g., carotenoids, chlorophyll, and polyphenols), can be effectively exploited to increase the quality and the stability of fats, oils, meat and fat-containing food products, preventing oxidation reactions [1] as well as for colouring, flavouring, and technological purposes (e.g., water/oil holding ability, air/water surface activity, and emulsifying capacity) [2]. Moreover, plant extracts could be used to fortify food products with polyphenols [3], whose consumption has been inversely associated with the risk of developing diseases linked to oxidative stress [4].

In this frame, the female inflorescence of hops (*Humulus lupulus* L.), known worldwide for its use in brewing production, represent a rich source of antioxidants and other compounds with biological activity. Since ancient times, hop has been used in folkloric medicine and aromatherapy for its numerous beneficial properties including anti-inflammatory, antiseptic, hypnotic, sedative, diuretic and antispasmodic ones [5,6], and its use has been also approved by the Committee on Herbal Medicinal Products (HMPC) of the European Medicines Agency, the German Commission E and the European Scientific Cooperative on Phytotherapy (ESCOP, 2003) to alleviate sleep disorders and mild mental and mood disorders (i.e., excitability, restlessness, anxiety) [6]. The multiplicity of beneficial effects exerted by hops are attributed to the high content of bioactive compounds and, in particular, to three main groups of secondary metabolites i.e., resins, essential oils, and polyphenols [7], whose presence and concentration depends on variety, harvest time and year of production [5,8].

With regards to polyphenols, four main chemical classes have been found in hops, i.e (I) flavan-3-ols, (II) flavonols, (III) phenolic carboxylic acids (derivatives of benzoic acid and cinnamic acid), and (IV) prenylflavonoids, which exhibit a strong protective action on human health thanks to antioxidant, immune-modulatory actions, anti-inflammatory, anticancer-related and antibacterial activity [5]. In particular, for xanthohumol, a prenylflavonoid characteristic of this plant, anti-infective effects against Gram-positive bacteria (e.g., *S. aureus*, *S. mutans*) and virus were observed, especially when combined with other molecules like the iso-α-acids [9] such as in hop extracts. However, the optimal recovery and preservation of these compounds to produce food-grade hop extracts, as for other plants, necessarily requires the selection of suitable solvents and extraction processes.

The technologies conventionally applied to produce plant extracts include solid-liquid extractions (SLE), such as percolation, maceration, and mechanical agitation extraction, which exploit leaching by solvent. However, solid-liquid extractions present some disadvantages that make their application quite uneconomical and not sustainable due to excessive consumption of time, energy, and polluting solvents [10]. This has led to deepening identification and development of innovative eco-friendly extraction methods such as supercritical fluid extractions (SFE). Unfortunately, SFE-based methods, besides their low energy input [11], have some shortcomings such as high establishment cost, need for highly specialized technical personnel, no scalability, and the selective nature of CO_2_, which is not suitable for the extraction of polar phenolics without the use of co-solvents [10]. For these reasons, recently, other non-conventional and green extraction techniques, including high hydrostatic pressure (HHP) and ultrasound-assisted extraction (UAE) have been also investigated to recover bioactive compounds from plants in order to obtain acceptable results in terms of both yields and environmental sustainability of the applied process [10]. UAE is an emerging non-thermal extraction, which allows the enhancement of solid/liquid mass transfers by acoustic cavitation induced into the liquid medium. In particular, it has been reported that low-frequency, high-power ultrasound is linked to increased cavitation during UAE and, consequently, higher extraction efficiency. Other advantages of using this technique includes simplicity, safety, versatility, rapidity, eco-friendliness, due to the reduced time, consumption of energy, and solvents [12].

HHP processing involves the application of high (from 100 to 800 MPa) isostatic hydraulic pressures (US Food and Drug Administration Centre for Food Safety and Applied Nutrition, 2000) and is generally applied for food processing and shelf-life extension due to their inactivating effect on pathogenic and spoilage microorganisms. However, beside these applications, HHP can also cause an increase of cytoplasmatic membrane permeability [13] and thus, enhance the mass transfer rates and diffusion of secondary metabolite from plant cells to the extraction solvent, allowing high bioactive extraction yield [14] with shorter extraction times.

Thus, with the aim to produce hop extracts rich in phytochemicals to use for the development of new, functional, and/or “free form” additives food products, in this study, the effectiveness of innovative (UAE and HHP) extraction methods was evaluated and compared with that of conventional (dynamic maceration) extractions. Hop extracts were characterised for the presence and concentration of single and total polyphenols, total carotenoid and chlorophyll content, and antiradical capacity. To discriminate the hop extracts and to highlight the interrelations among all the variables analysed and extraction parameters (technique, temperature, and time), the whole dataset was subjected to PLS-DA (Partial Least-Squares Discriminant Analysis) and PLS-R (Partial Least-Squares Regression) analysis.

## 2. Materials and Methods

### 2.1. Plant Material

A batch of hop cones (*H. lupulus*) cv. Cascade grown in Abruzzo (Italy) and harvested in 2018 was used for all the experiments. Freshly harvested cones were dried at 40 °C to a moisture content of less than 12%, vacuum-packed in high-barrier plastic bags and stored at −40 °C until use.

### 2.2. Chemicals

All used reagents were purchased from Sigma-Aldrich (Steinheim, Germany). Organic solvents used for chromatography were of HPLC ultragradient grade (Sigma-Aldrich, Milan, Italy). The water used throughout the experiments was previously purified in a Milli-Q system (Millipore, Milan, Italy). Polytetrafluoroethylene (PTFE) membrane filters (0.45-μm pore size) from Pall (Pall Corporation, Michigan, USA) were used for filtration of both mobile phases and hop extracts.

### 2.3. Preliminary Operations

Before extraction, dried hop cones were ground to a fine powder according to Inui et al. [8] and packed in Polyamide/Polyethylene/Polyethylene (PA/PE/PE) plastic bags and kept protected from light and humidity until extraction.

### 2.4. Preliminary Experiments

**Figure 1:**
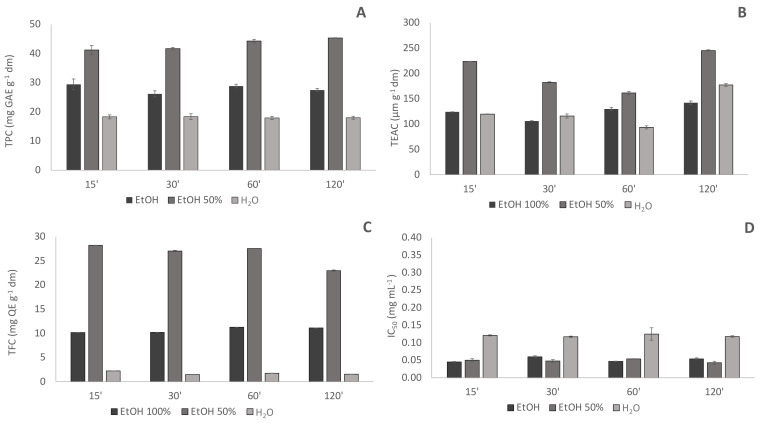
Comparison of phenolic content and antioxidant capacity of pure ethanol (EtOH); water (H_2_O) and ethanol water 50:50 (*v*/*v*) (EtOH 50%) hop extracts obtained by conventional extraction at 60 °C (CONV 60 °C); (**A**) TPC: Total Phenolic Content (mg GAE g^−1^ dm); (**B**) TEAC: Trolox Equivalent Antioxidant Capacity (µmol g^−1^ dm); (**C**) TFC Total Flavonoids Content (mg QE g^−1^ dm); (**D**) antiradical capacity express as Inhibitory Capacity IC50 (mg mL^−1^); 15′, 30′, 60′, 120′: extraction times in minutes.

**Figure 2:**
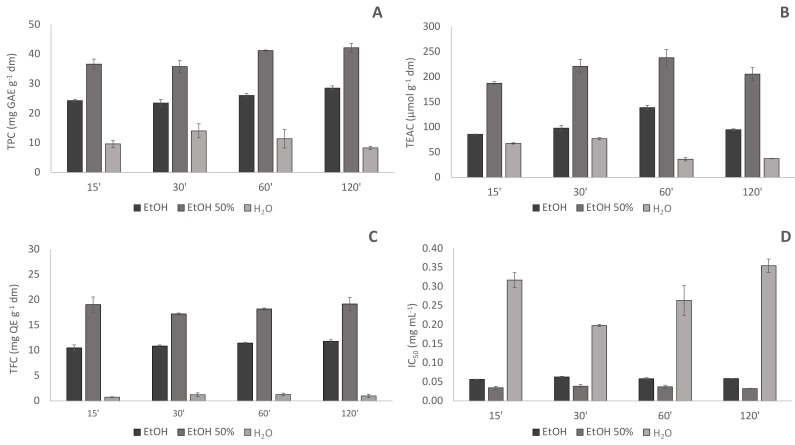
Comparison of phenolic content and antioxidant capacity of pure ethanol (EtOH); water (H_2_O) and ethanol water 50:50 (*v*/*v*) (EtOH 50%) hop extracts obtained by high power ultrasounds (HPUS); (**A**) TPC: Total Phenolic Content (mg GAE g^−1^ dm); (**B**) TEAC: Trolox Equivalent Antioxidant Capacity (µmol g^−1^ dm); (**C**) TFC Total Flavonoids Content (mg QE g^−1^ dm); (**D**) antiradical capacity express as Inhibitory Capacity IC50 (mg mL^−1^); 15′, 30′, 60′, 120′: extraction times in minutes.

The solvent, and matrix-solvent ratio were selected based on a set of preliminary tests. In particular, for each solvent, three different matrix-solvent ratios (1:10; 1:20; 1:50 *w*/*v*) were investigated and among them, the ratio 1:50 allowed the highest recovery of antioxidant compounds (*data not shown*). Selecting this matrix-solvent ratio, the extraction efficiency of pure ethanol, water, and ethanol:water 50:50 *v*/*v* towards antioxidant compounds was thus compared using as extraction methods both dynamic maceration at 60 °C and high power ultrasounds for times ranging from 15 to 120 min. Based on the obtained results (Figure 1 and Figure 2), all the subsequent investigations were carried out using ethanol:water 50:50 (*v*/*v*) as extraction solvent, and a matrix-solvent ratio of 1:50 (*w*/*v*).

### 2.5. Conventional Extraction by Dynamic Maceration

Conventional (CONV) extractions were carried out under constant stirring (300 rpm), at 25 °C (CONV 25 °C) and 60 °C (CONV 60 °C) for 15, 30, 60 and 120 min. Both CONV 25 °C and CONV 60 °C extractions were performed in triplicate.

### 2.6. Innovative Extractions

Innovative (INN) extractions were carried out by ultrasound and high hydrostatic pressure technologies. Before extraction, the hop powder and the solvent were packed into PA/PE/PE plastic (50 µm thick film) bags in absence of air.

#### 2.6.1. UAE Extraction

Ultrasounds-assisted extractions (UAE) were carried out at 25 °C for 15, 30, 60 and 120 min using two different thermally-controlled ultrasonic tools: (i) a low power (100 W, 50 kHz) ultrasound (US) bath (LABSONIC LBS1 -3, FALC, Bergamo, Italy) and (ii) a high power (800 W) ultrasound (HPUS) bath (Waveco^®^, Next Cooking Generation, Milano, Italy). Both US and HPUS extractions were performed in triplicate.

#### 2.6.2. HHP Extraction

HHP extraction was performed using an industrial-scale equipment (Avure HPP AV-10, JBT, Chicago, IL, USA). The bags with the samples were placed into a hydrostatic pressure vessel and pressure was raised up to 600 MPa. The pressure holding time was 5 min, and the overall treatment duration was of ca. 8 min. Water was used as a filling medium in the HHP vessel with an initial temperature of 3 °C. Considering an adiabatic temperature increase of 3 °C/100 MPa, the vessel water temperature, at the fixed process pressure (600 MPa), was estimated to be ≤21 °C. The HHP extraction was performed in triplicate.

CONV and INN extracts were centrifuged at 4000 rpm (2470× *g*) for 10 min at 4 °C, then the supernatants were filtered with a nylon filter (0.45 µm) and stored at −40 °C until analysis.

### 2.7. Total Phenolic Content and Antioxidant Capacity

Total phenolic content (TPC) and the ABTS radical scavenging activity were determined using the Folin-Ciocalteau reagent and the ABTS radical cation decolorization assay as described by Santarelli et al. [15], and expressed as gallic acid equivalent (GAE) and Trolox equivalent antioxidant capacity (TEAC; μmoles of Trolox equivalents *per* g of dry matter), respectively.

DPPH radical scavenging activity was performed according to Pellegrini et al. [16], and data were expressed as IC_50_ (mg mL^−1^), corresponding to the concentration needed to cause 50% of the antiradical effect, thus lower values account for higher antiradical capacity.

All determinations were performed in triplicate.

### 2.8. Total Flavonoid Content

Total flavonoid content (TFC) was determined according to Kowalczyk et al. [7], without modifications. Results were expressed as mg of quercetin equivalent (QE) g^−1^ dm.

The assay was carried out in triplicate on each extract.

### 2.9. Content of Chlorophyll α, Chlorophyll β and Total Carotenoid

The content of chlorophyll *α* (chl *α*) and *β* (chl *β*), and total carotenoids were determined according to Kobus-Cisowska et al. [17] without modifications. Results were given in mg g^−1^.

### 2.10. Content of Phenolic Compounds and Xanthohumol by HPLC Analysis

The content of single phenols and xanthohumol was evaluated by HPLC analysis by using a chromatographic system (Agilent 1100 series, Agilent, Italy) equipped with a photodiode array detector (DAD; Agilent Technologies, Milan, Italy), according to Carbone et al. [18], without modifications. All analytical data were processed by the data management software system (ChemStation 32.1, Agilent Technologies). Six-point calibration curve based on external standard solutions (0–100 ppm) were used for quantification. Results were expressed as mg g^−1^ of dried hops.

### 2.11. Statistical Analysis

Data were reported as mean and standard deviation and analysed by one way ANOVA analysis. Significant differences between means were calculated by LSD post hoc test at a level *p* < 0.05.

Data collected on the CONV and UAE extracts were additionally processed by multifactorial ANOVA to highlight, for the formers, the single and combined effects of the extraction temperature (T) and time (t), and for the latter the single and combined effects of the extraction method (EM) and time (t).

To study the data structure, partial least squares-discriminant analysis (PLS-DA) and partial least-squares regression (PLS-R) were computed. These methods were applied on complete standardized dataset (34 samples × 21 variables) to retrieve all relevant information systematically. In PLS-DA analysis cross-validation was conducted through general Jackknife method using 5 groups that one by one were removed in order to recompute the model. Qi^2^ criterion was used to determine if the contribution of latent variables (LV) to all dependent variables were significant. PLS-R (PLS1) was performed on the complete dataset and extraction time was chosen as unique continuous dependent variable of the regressions. In all PLS depicted a Q^2^ > 0.5 was obtained as an index of good stability of the models. Variable importance in projection (VIP) greater than one was used as selection method in order to investigate the most important variables capable of discriminating different extraction conditions (method and time). All statistical analyses were performed using XLSTAT 2021 (Addinsoft, Paris, France).

## 3. Results and Discussion

### 3.1. Phenolic Profile of Hop Extracts

In Table 1, the phenolic composition of the hop extracts obtained by the different extraction methods is reported. According to literature, different classes of phenolic compounds were detected, including hydroxybenzoic acids (gallic, ellagic, protocatechuic, syringic, *p*-hydroxybenzoic acid), hydroxycinnamic acids (chlorogenic, chicoric, *p*-coumaric, ferulic and caffeic acid), flavanols (catechin and epigallocatechin), rutin and the prenylated chalcone xanthohumol [8,18]. Conversely, no peaks related to flavonoids usually found in hops [8], such as quercetin and kaempferol, could be identified by using both retention times and absorption spectra of reference compounds.

In the hop extracts, the most abundant compounds found were catechin and xanthohumol with a concentration ranging, depending on the method of extraction used, from 1.22 mg g^−1^ to 2.70 mg g^−1^ and from 1.09 mg g^−1^ to 2.67 mg g^−1^, respectively. Rutin and chlorogenic acid varied between 0.612 and 0.877 mg g^−1^, while *p*-hydroxybenzoic, protocatechuic, syringic and ellagic acids were determined in quantities ranging from about 0.262 to 0.654 mg g^−1^. Other polyphenols i.e., epigallocatechin, *p*-coumaric, caffeic and ferulic acids were found in minor concentrations and at very different extent depending on the type of extraction applied.

The direct comparison of these results with others reported in literature is not possible since, to the best of the authors’ knowledge, available data of single and total polyphenols content in hops are referred to wild hops [18] or different hop variety [7] and/or different extraction solvents [8,17], factors that affect the phenolic pattern of the hop extracts both qualitatively and quantitatively.

By comparing the conventional extractions at 25 and 60 °C, and US and HPUS extraction, it can be observed that the conventional methods led to a higher extraction efficiency of xanthohumol while UAE, in general, promoted the extraction of rutin and protocatechuic acid. Gallic acid was found only in UAE extracts and for extraction times lower than 120 min, while chicoric acid was detected only in US extracts. Conventional extraction at 25 °C compared to that at 60 °C and to UAE extractions generally showed the poorest extraction efficiency towards chlorogenic, caffeic, and *p*-coumaric acid.

In order to analyse the single and combined effect of the process variables on the polyphenol extraction efficiency, the single and total polyphenol content of the extracts obtained by conventional and UAE extractions were processed by factorial ANOVA. The factors analysed were respectively the extraction temperature (T) and time (t) for the former, and the ultrasound extraction method (EM) and the time (t) for the latter, and the results are shown in Table S1 (Supplementary Materials).

In conventional extractions, the extraction temperature positively influenced (*p* < 0.01) the content of all the detected polyphenols. The extraction time (t) had a positive effect on the extraction of syringic, ellagic, chlorogenic, *p*-hydroxybenzoic, *p*-coumaric, ferulic and caffeic acid, rutin and epigallocatechin, a negative effect on that of catechin, while it had no effect on the extraction of protocatechuic acid and xanthohumol. The negative effect of the extraction time on catechin content can be attributed to the tendency of this molecule to degrade during prolonged extraction, as also observed by Perva-Uzulanic et al. [19], in green tea extracts due to oxidation reactions and polymerization in C–C or C–O–C linked dimers [19,20]. The combined effect of T × t positively influenced the total polyphenol content by increasing the extraction of all the detected compounds except for the syringic and ellagic acid, and catechin.

Regarding the UAE extracts, the extraction methods (EM), i.e., US and HPUS, did not influence (*p* > 0.05) the total polyphenol content and the extraction of protocatechuic acid, catechin, caffeic acid, while it significantly and positively influenced that of the other polyphenols. In particular, the content of gallic, *p*-hydroxybenzoic, syringic, ellagic, chlorogenic, *p*-coumaric, ferulic acid, rutin and epigallocatechin was higher in the US extracts, while xanthohumol in the HPUS ones. Conversely to HPUS, US allowed also to extract a small amount of cichoric acid (dicaffeoyl D-tartaric acid), known for its high bioactive potential [21].

In the UAE methods, the extraction time (t) positively affected both the total polyphenol content and the single phenol content of hop extracts, with the exception of catechin, which, as also observed in the conventional extractions, was impaired by the extraction time, and of the protocatechuic acid for which no significant effect was observed. These results agree with those reported by Ma et al. [22] who found a positive effect of both ultrasound frequency and time on the extraction of polyphenols from Satsuma mandarin (*Citrus unshiu* Marc.) peels and citrus peel.

Concerning the hop extract obtained by HHP extraction (Table 1), despite the lowest extraction time, it showed a content of hydroxybenzoic acids similar to the extract CONV 25 °C 30 min and quantities of hydroxycinnamic acids, rutin and xanthohumol similar to those found in the extract US 60 min. However, in respect to extracts obtained by UAE or by conventional extraction for the longest treatment times, an overall lower amount of polyphenol compounds was determined. These results are in disagreement with what was observed by Jun [23] on green tea leaves. The author, in fact, investigating the effect of HHP on the extraction of polyphenols using process and extraction conditions similar to those adopted in this study (600 MPa for 4 min; ethanol 50% *v*/*v*), found no difference in the extraction efficiency compared to the other conventional (agitation at room temperature for 20 h) and innovative (UAE for 90 min) extraction methods.

These discrepancies in the HHP extraction efficiency could be related to the different composition, structural properties, and particle size and of the plant matrices under investigation, which are factors influencing the matrix-solvent interactions and mass transfers of phenolic compounds into the extraction solvent.

### 3.2. Hop Extracts Antioxidants and Antioxidant Capacity

In Table 2 data of the total phenolic (TPC), total flavonoid (TFC), chlorophyll *α* (chl *α*), chlorophyll *β* (chl *β*), and total carotenoid (TCC) content, as well as the antiradical capacity, evaluated by both the ABTS and DPPH assays, of the hop extracts obtained by the different extraction methods, are reported.

Depending on the extraction process, TPC varied between 34.2 and 48.5 mg GAE g^−1^, according to what reported by Kowalczyk et al. [7] and by Liu et al. [24], respectively for 50% and 100% ethanolic extracts of different hop varieties, while Wu et al. [25], analysing 55% ethanolic extracts collected from hop pellets, obtained higher TPC values. By comparing the total polyphenol content determined on hop extracts by HPLC and TPC analyses it is possible to observe as the obtained values for each extract are very different among them, this because the method used for ‘TPC’ measures the capacity of hop extracts to reduce the Folin-Ciocalteu’s reagent and thus is an index of the reducing power of the extract [26].

TFC ranged between 13.1 and 28.2 mg QE g^−1^ in agreement with what indicated by Wu et al. [25] while Kowalczyk et al. [7] for 50% ethanolic extracts of Magnum and Marynka varieties and Mafakheri & Hamidoghli [27] for 75% (*w*/*w*) ethanolic extracts of wild hops reported higher values. Finally, the chl *α* ranged from 0.363 to 1.19 mg g^−1^, chl *β* ranged from 0.31 to 1.6 mg g^−1^, and TCC ranged from 1.14 to 2.20 mg g^−1^, and these values were higher than those reported by Kobus-Cisowska, et al. [17].

As concerns the antiradical activity, TEAC varied between 173 and 271 µmol g^−1^ (dm) and IC_50_ from 0.032 to 0.081 mg mL^−1^. These results are lower than that measured by Kobus-Cisowska et al. [17] in 40 % (*w*/*w*) ethanol extracts of Magnum, Marynka and Lubelsky varieties, while the DPPH radical scavenging capacity was higher compared to that reported by Mafakheri and Hamidoghli [27].

By comparing the TPC, TFC and the antiradical capacity of hop extracts with those of other categories of spices and foods generally considered to be rich sources of antioxidant compounds [28,29,30] it is possible to note this plant represents an extraordinary source of polyphenols and other antioxidant compounds.

To analyse the single and combined effect of extraction temperature (T) and time (t) on the content of each class of antioxidant compounds and the antiradical activity of hop extracts obtained by conventional extraction methods, data were processed by factorial ANOVA, and results are reported in Table S2 (Supplementary Materials).

In particular, the extraction at 60 °C compared to that carried out at 25 °C determined, on average, the increase of TPC, TFC and of TCC respectively of 25%, 40% and 52%, and tripled the content of chl *α* and chl *β* (Table 2). Regarding the antiradical capacity, according to Thoo et al. [31] the use of high extraction temperature had a positive effect on the DPPH radical scavenging capacity of the hop extracts, whilst the effect on the ABTS radical scavenging capacity varied dependently on the time of extraction. Any differences between results obtained using the two assays may be due to the different polarity of the reaction solvent and different reaction times of antioxidants with ABTS and DPPH radicals since some compounds can react almost instantaneously whilst others are slow reacting antioxidants [32]. Moreover, it is possible that some compounds that have ABTS scavenging activity, after reaction may assume structures with a higher antioxidant capacity and react again with ABTS [33,34].

The increase of TPC and TFC with the increase of the extraction temperature is in accordance with the results presented by other authors on different plant matrices [31,35] and attributed to various factors, including (I) the breakdown and release of free phenols otherwise covalently bound with plant cell insoluble polymers (protein-phenol and polysaccharide-phenol interactions), (II) increased phenolic solubility, (III) the increase of mass transfer rate; and therefore of the extraction rate, and (IV) the reduction of solvent viscosity and surface tension [35,36,37]. The higher extractability of the phenolic compounds from the matrix as well as the formation of new compounds with antioxidant activity were responsible for the increase of the antioxidant capacity of hop extracts [38]. 

As regards the extraction time (t), it had a positive effect on all tested assays with the exception of TFC, for which the prolonged extractions caused a decrease of the flavonoid content. As shown by HPLC data (Table 1) this could be due to the decrease of catechin content and, eventually, of other not identified compounds.

The different effect of extraction times on TPC and TFC may also be due to the different degree of polymerization of phenols, their solubility and interaction with other matrix constituents, which is reflected in a difference in the time needed to achieve a balance between the solution in the solid matrix and in the bulk solution [31].

Finally, the combined effect of T × t significantly affected the extraction of all the detected compounds apart from the carotenoid content (Table S2 Supplementary Materials).

By comparing the functional properties of hop extracts obtained by conventional and ultrasound-assisted extractions, it can be noted that, in general, the former showed the highest TFC values, while the latter were characterized by the highest antiradical capacity. The ultrasound extraction allows, in fact, to increase the swelling and hydration of the dry matrices and therefore to improve diffusion processes and the mass transfers through the cell walls, leading to higher extraction yields [36,39]. Moreover, the hydroxylation of flavonoids at the ortho, meta- or para-radical level by the hydroxyl radicals generated during the sonication process may have contributed to the increase in antiradical activity [37].

In order to analyse the single and combined effect of the ultrasound extraction method (EM) and of the extraction time (t) on the antioxidant content and antiradical properties of hop extracts, data were processed by multifactorial ANOVA, and the related results are shown in Table S2 (Supplementary Materials).

It is possible to highlight that the extraction method (EM) significantly influenced the properties of the hop extracts. In particular, the US extracts compared to the HPUS ones showed a greater TPC and TEAC as well as a higher chl *α*, chl *β*, and TCC and a lower TFC and DPPH antiradical capacity. These differences could be due to the lower ultrasound frequency of the US system compared to the HPUS one. In fact, as the frequency of ultrasound increases, the production and intensity of cavitation in the liquid decreases: at high frequencies, the compression-rarefaction cycles may be too short to allow the growth of cavitation bubbles; while at low frequencies, the transient cavitation bubbles are relatively less numerous but with a large diameter, promoting physical rather than chemical effects [39], which determine the increase of the extraction efficiency [40]. The different results between the DPPH and ABTS assays can be explained by the different mechanism of the reactions involved in the measurement of the antiradical capacity. ABTS cation radical reactions involve, in fact, the transfer of electrons and occur at a much faster rate than those for DPPH radicals, whose degree of discoloration is attributed to the ability to donate hydrogen [41].

The HHP extraction provided similar results as those of conventional ones carried out at 25 °C, but lower values than all the others under investigation, with respect to all the detected compounds (Table 2). This result is in disagreement with what observed by Prasad et al. [42] on longan fruit pericarp. Further studies are necessary in order to investigate the influence of the extraction time on the extraction of bioactive compounds from hop, although several studies have observed that as the extraction time increases, no significant increases in yields of bioactive compounds are noticeable [14,43].

### 3.3. Supervised Multivariate Analysis

To better understand the interrelations among all the variables analysed and extraction parameters, the whole dataset was subjected to PLS–DA and PLS–R analysis. In particular, to check if different extraction methods and time can discriminate different hop extracts, PLS–DA and PLS–R were respectively computed, and for PLS–DA analysis only the variables with a VIP greater than one are mentioned and discussed.

**Figure 3:**
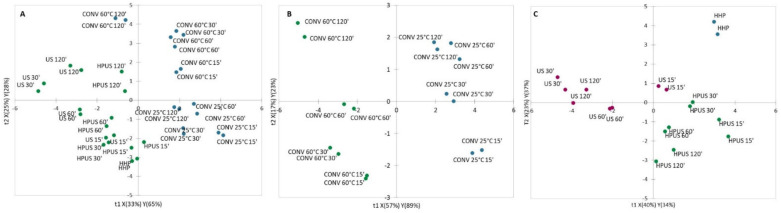
Score plots along the two of the PLS–DA model. Predictor variables were: (**A**) CONV (CONV 25 °C and CONV 60 °C) and INN (US, HPUS, HHP); (**B**) CONV 25 °C and CONV 60 °C; (**C**) US, HPUS, HHP extractions. CONV 25 °C: conventional extraction at 25 °C; CONV 60 °C: conventional extraction at 60 °C; HPUS: high power ultrasounds; US: low power ultrasounds; HHP: high hydrostatic pressure; 15’, 30’, 60’, 120’: extraction times in minutes.

A first PLS–DA was computed using CONV and INN extractions as qualitative variables and all bioactives were used as explanatory variables. The model depicted two significant LVs and explained 84% and 58% of the total variance of Y and X. The model permitted to correctly classify all hop extracts with a classification rate of 100% as confirmed by the score plot (Figure 3A), where a clear discrimination can be seen between conventional and innovative extractions along the first LV.

Xanthohumol was a variable with a positive β-coefficient, which characterized CONV extracts, whereas TFC, rutin, gallic, protocatechuic and *p*-coumaric acid were variables with a negative β-coefficient, which characterized INN extracts.

In order to understand and better visualize the discrimination within conventional and innovative extractions, two additional PLS–DA on the two sub-groups were computed.

The PLS–DA model with CONV 25 °C and CONV 60 °C extractions (first sub-group) as qualitative variables depicted two significant LVs and explained 97% and 74% of Y and X of total variance. The model permitted to correctly classify all hop extracts with a classification rate of 100% as confirmed by the score plot (Figure 3B), where a clear discrimination can be seen within conventional extractions at different temperature along the first LV. Since all the VIPs had a negative β-coefficients i.e., TFC, TPC, chl *α* chl *β*, TCC, DPPH, xanthohumol, epigallocatechin, *p*-coumaric and syringic acid, the CONV 60 °C method confirmed to extract higher amount of bioactives with respect to CONV 25 °C as also demonstrated by factorial ANOVA analysis previously discussed.

The PLS–DA model with HPUS, US and HHP extractions (second sub-group) as qualitative variables depicted two significant LVs and explained 75% and 57% of Y and X of total variance. The model permitted to correctly classify all hop extracts with a classification rate of 100% as confirmed by the score plot (Figure 3C), where a discrimination can be seen within innovative extraction methods.

The first LV permitted to separate clearly HPUS from US except for US 15′ samples, while the second LV permitted to discriminate HHP extracts.

In particular, TFC and xanthohumol (w1 positive) characterized HPUS extracts; TPC, antiradical capacity measured by both ABTS and DPPH assays, chl *α*, chl *β* and TCC (w1 negative) characterized US; TPC, antiradical capacity measured by both ABTS and DPPH assays, and TCC (w2 positive) characterized HHP extracts.

Lastly, a PLS–R was computed where extraction time represented continuous predictor variable. The model depicted two significant LVs and explained 81% and 57% of the total variance of Y and X. Gallic, *p*-coumaric acid and catechin decreased with increasing extraction time (negative β-coefficient) on the contrary hydroxybenzoic, ellagic, caffeic and ferulic acid decreased with increasing extraction time (positive β-coefficient).

**Figure 4:**
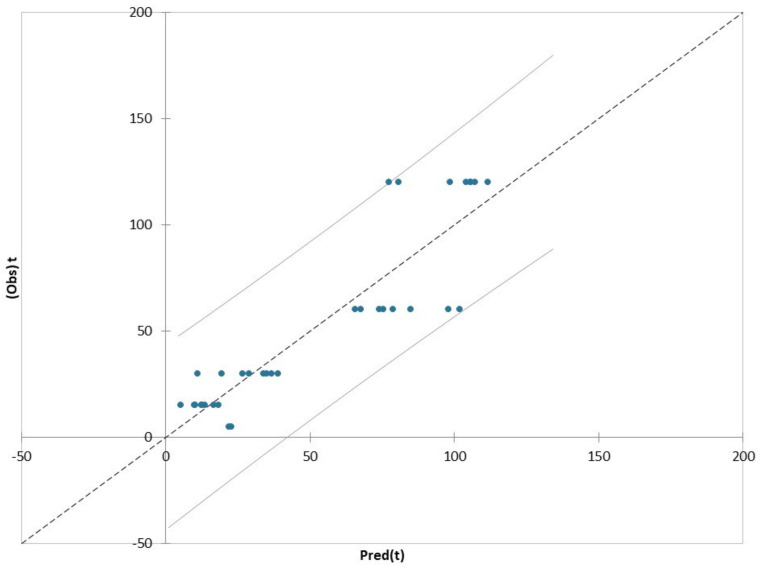
PLS regression between predicted and observed values on t (time) variable with confidence intervals using the complete dataset.

Figure 4 shows PLS regression plot between predicted and observed values on t (time) variable with confidence intervals using the complete dataset. The model depicted a clear separation into two main groups characterized by extraction time <30 min (i) and >60 min (ii). The extracts with higher residuals in prediction were those obtained after 60 and 120 min (Table S3 Supplementary Materials), in particular CONV 60 °C 60′ and CONV 25°C 120′ extracts. The PLS–R model predicted extraction times on average of 100 min for the CONV 60 °C 60′ samples, indicating a high extraction efficiency at this time. On the contrary, for CONV 25 °C 120′ sample, the model predicted extraction times of about 78 min, indicating a minor extraction efficiency of this extraction. Probably due to a saturation effect, the extraction for 120 min is not particularly efficient for the recovery of bioactive compounds from hop.

Furthermore, although the hop extract obtained by HHP technique showed lower values than all the others under investigation, with respect to all the detected compounds (Table 1 and Table 2), the PLS–R model overestimated the extraction time on average of 20 min (predicted extraction time) instead of 5 min (observed extraction time) (Table S3 Supplementary Materials), indicating a greater extraction efficiency with respect to the short extraction time.

Overall, among all the extraction processes under investigation, CONV 60 °C and US for 30 min allowed the highest polyphenol extraction with the former being more selective with respect to xanthohumol and the latter to gallic and chicoric acids. However, as also pointed out by Ma et al. [21], the selection of the best method and time for the extraction of hop polyphenols along with its optimal conditions should be based on the specific phenolic compound of interest.

## 4. Conclusions

The experimental results indicate that the efficiency in bioactive recovery from hop is strongly influenced by the extraction conditions used, in terms of extraction method and time. Statistical analyses were effective in showing the best technological conditions to produce hop extracts with higher functional properties.

Among the extraction methods under study, both low-power ultrasound extraction (US) and conventional extraction carried out at high temperature (CONV 60 °C) showed the highest content of bioactive compounds. Overall, considering the short extraction time and low energy consumption, and high recovery of bioactive compounds, the US represents a valid and green extraction method to be used for rapid extraction of active compounds from hop.

To our knowledge, this study could be of great relevance, as optimal recovery of phytochemicals from hop by food-grade and green extractions provide an enormous potential for the development and production of functional and clean label food products according to the current needs of industry and consumers.

## Figures and Tables

**Table 1:** Phenolic composition (mg g^−1^) of different hop extracts determined by high-performance liquid chromatography (mean ± standard deviation).

		**mg g^−1^**							
**Extraction Method**	**Time (min)**	**GA**	***p*OH-B**	**SyrA**	**EllA**	**ProCA**		**Cat**	**EGC**
CONV 25 °C	15	n.d.	n.d.	0.320 ± 0.008 ^i^	0.262 ± 0.008 ^g^	0.433 ± 0.048 ^ef^		2.38 ± 0.07 ^de^	0.095 ± 0.006 ^ef^
	30	n.d.	0.452 ± 0.005 ^e^	0.374 ± 0.013 ^h^	0.290 ± 0.003 ^fg^	0.519 ± 0.009 ^bc^		2.19 ± 0.05 ^f^	0.087 ± 0.001 ^fgh^
	60	n.d.	0.519 ± 0.005 ^d^	0.380 ± 0.001 ^gh^	0.485 ± 0.068 ^c^	0.441 ± 0.005 ^def^		1.22 ± 0.02 ^l^	0.086 ± 0.004 ^fgh^
	120	n.d.	0.531 ± 0.002 ^d^	0.398 ± 0.010 ^fgh^	0.441 ± 0.014 ^d^	0.462 ± 0.001 ^cdef^		1.34 ± 0.02 ^il^	0.083 ± 0.004 ^fgh^
CONV 60 °C	15	n.d.	n.d.	0.416 ± 0.001 ^efgh^	0.315 ± 0.001 ^f^	0.555 ± 0.030 ^ab^		2.74 ± 0.09 ^a^	0.105 ± 0.009 ^de^
	30	n.d.	0.529 ± 0.042 ^d^	0.515 ± 0.042 ^bc^	0.361 ± 0.005 ^e^	0.418 ± 0.006 ^f^		2.50 ± 0.05 ^cd^	0.146 ± 0.003 ^a^
	60	n.d.	0.614 ± 0.005 ^bc^	0.482 ± 0.006 ^cd^	0.628 ± 0.025 ^a^	0.525 ± 0.001 ^bc^		1.44 ± 0.05 ^hi^	0.124 ± 0.008 ^b^
	120	n.d.	0.634 ± 0.002 ^ab^	0.544 ± 0.009 ^ab^	0.570 ± 0.023 ^b^	0.558 ± 0.001 ^ab^		1.61 ± 0.04 ^g^	0.147 ± 0.005 ^a^
HPUS	15	0.079 ± 0.001 ^d^	0.474 ± 0.001 ^e^	0.411 ± 0.026 ^efgh^	0.300 ± 0.013 ^fg^	0.506 ± 0.024 ^bcde^		2.68 ± 0.09 ^ab^	0.094 ± 0.002 ^ef^
	30	0.087 ± 0.001 ^bc^	0.466 ± 0.009 ^e^	0.419 ± 0.047 ^efgh^	0.292 ± 0.001 ^fg^	0.566 ± 0.050 ^ab^		2.72 ± 0.13 ^ab^	0.117 ± 0.001 ^bc^
	60	0.086 ± 0.001 ^c^	0.589 ± 0.032 ^c^	0.419 ± 0.014 ^efg^	0.490 ± 0.011 ^c^	0.580 ± 0.104 ^ab^		1.26 ± 0.03 ^l^	0.079 ± 0.002 ^h^
	120	n.d.	0.589 ± 0.015 ^c^	0.481 ± 0.024 ^cd^	0.494 ± 0.025 ^c^	0.515 ± 0.020 ^bcd^		1.52 ± 0.07 ^gh^	0.108 ± 0.009 ^cd^
US	15	0.085 ± 0.003 ^c^	0.529 ± 0.010 ^d^	0.453 ± 0.021 ^de^	0.297 ± 0.001 ^fg^	0.548 ± 0.041 ^ab^		2.37 ± 0.00 ^e^	0.088 ± 0.002 ^fgh^
	30	0.099 ± 0.002 ^a^	0.591 ± 0.010 ^c^	0.575 ± 0.007 ^a^	0.379 ± 0.007 ^e^	0.616 ± 0.010 ^a^		2.60 ± 0.05 ^bc^	0.141 ± 0.005 ^a^
	60	0.091 ± 0.003 ^b^	0.640 ± 0.007 ^ab^	0.439 ± 0.024 ^ef^	0.521 ± 0.001 ^c^	0.509 ± 0.012 ^bcd^		1.33 ± 0.06 ^il^	0.086 ± 0.003 ^fgh^
	120	n.d.	0.654 ± 0.002 ^a^	0.515 ± 0.002 ^bc^	0.522 ± 0.001 ^c^	0.513 ± 0.049 ^bcd^		1.60 ± 0.00 ^g^	0.122 ± 0.001 ^b^
HHP	5	n.d.	0.472 ± 0.021 ^e^	0.401 ± 0.001 ^fgh^	0.295 ± 0.005 ^fg^	0.544 ± 0.005 ^ab^		2.34 ± 0.05 ^e^	0.091 ± 0.004 ^fg^
**on**		**mg g^−1^**							
**Extraction Method**	**Time (min)**	**ChlA**	**ChicA**	**CafA**	**FerA**	**pCuA**	**Rut**	**XAN**	**TOT**
CONV 25 °C	15	0.466 ± 0.001 ^m^	n.d.	n.d.	n.d.	n.d.	0.653 ± 0.024 ^i^	1.92 ± 0.09 ^d^	6.53 ± 0.26 ^ilm^
	30	0.484 ± 0.004 ^lm^	n.d.	0.012 ± 0.002 ^de^	0.012 ± 0.005 ^ef^	0.029 ± 0.004 ^hi^	0.656 ± 0.029 ^i^	1.73 ± 0.16 ^ef^	6.83 ± 0.27 ^ghi^
	60	0.503 ± 0.006 ^il^	n.d.	0.008 ± 0.005 ^e^	0.022 ± 0.019 ^def^	0.014 ± 0.010 ^i^	0.612 ± 0.002 ^l^	1.88 ± 0.01 ^de^	6.17 ± 0.12 ^n^
	120	0.520 ± 0.001 ^hi^	n.d.	0.020 ± 0.001 ^cd^	0.015 ± 0.013 ^ef^	0.014 ± 0.007 ^i^	0.770 ± 0.010 ^g^	1.86 ± 0.08 ^de^	6.46 ± 0.01 ^lmn^
CONV 60 °C	15	0.525 ± 0.013 ^hi^	n.d.	0.010 ± 0.004 ^de^	0.021 ± 0.006 ^def^	0.114 ± 0.001 ^de^	0.727 ± 0.005 ^h^	2.34 ± 0.10 ^bc^	7.87 ± 0.05 ^cd^
	30	0.532 ± 0.007 ^ghi^	n.d.	0.026 ± 0.003 ^bc^	0.029 ± 0.010 ^cde^	0.159 ± 0.020 ^b^	0.681 ± 0.011 ^i^	2.67 ± 0.01 ^a^	8.57 ± 0.01 ^a^
	60	0.902 ± 0.003 ^a^	n.d.	0.034 ± 0.003 ^b^	0.054 ± 0.005 ^bc^	0.046 ± 0.001 ^h^	0.739 ± 0.005 ^gh^	2.19 ± 0.02 ^c^	7.77 ± 0.13 ^cd^
	120	0.580 ± 0.008 ^cd^	n.d.	0.055 ± 0.002 ^a^	0.097 ± 0.021 ^a^	0.104 ± 0.009 ^ef^	0.859 ± 0.009 ^ab^	2.49 ± 0.15 ^b^	8.25 ± 0.02 ^ab^
HPUS	15	0.531 ± 0.023 ^hi^	n.d.	0.018 ± 0.004 ^cd^	0.009 ± 0.02 ^ef^	0.109 ± 0.009 ^de^	0.767 ± 0.032 ^fg^	1.66 ± 0.15 ^fg^	7.64 ± 0.04 ^de^
	30	0.563 ± 0.024 ^defg^	n.d.	0.032 ± 0.001 ^b^	0.023 ± 0.001 ^def^	0.150 ± 0.005 ^b^	0.814 ± 0.020 ^cd^	1.41 ± 0.02 ^hi^	7.66 ± 0.29 ^d^
	60	0.547 ± 0.026 ^efgh^	n.d.	0.033 ± 0.003 ^b^	0.033 ± 0.023 ^def^	0.030 ± 0.007 ^hi^	0.785 ± 0.001 ^def^	1.44 ± 0.01 ^h^	6.36 ± 0.10 ^nm^
	120	0.789 ± 0.001 ^b^	n.d.	0.057 ± 0.008 ^a^	0.089 ± 0.019 ^a^	0.078 ± 0.019 ^g^	0.789 ± 0.005 ^def^	1.65 ± 0.04 ^fg^	7.15 ± 0.14 ^g^
US	15	0.577 ± 0.024 ^cde^	n.d.	n.d.	0.017 ± 0.005 ^def^	0.142 ± 0.015 ^bc^	0.736 ± 0.001 ^h^	1.49 ± 0.02 ^gh^	7.33 ± 0.02 ^ef^
	30	0.565 ± 0.026 ^cdef^	0.162 ± 0.002 ^a^	0.049 ± 0.012 ^a^	0.078 ± 0.002 ^ab^	0.199 ± 0.001 ^a^	0.783 ± 0.003 ^def^	1.23 ± 0.13 ^l^	8.08 ± 0.24 ^bc^
	60	0.567 ± 0.001 ^cdef^	0.011 ± 0.001 ^c^	0.033 ± 0.001 ^b^	0.042 ± 0.006 ^cd^	0.126 ± 0.006 ^cd^	0.831 ± 0.001 ^bc^	1.24 ± 0.00 ^il^	6.45 ± 0.11 ^mn^
	120	0.597 ± 0.007 ^c^	0.117 ± 0.005 ^b^	0.057 ± 0.005 ^a^	0.094 ± 0.005 ^a^	0.087 ± 0.001 ^fg^	0.877 ± 0.009 ^a^	1.23 ± 0.01 ^l^	6.98 ± 0.02 ^gh^
HHP	5	0.538 ± 0.015 ^fgh^	n.d.	0.026 ± 0.007 ^bc^	0.014 ± 0.007 ^ef^	0.119 ± 0.012 ^de^	0.801 ± 0.021 ^cde^	1.09 ± 0.05 ^l^	6.73 ± 0.09 ^hil^

CONV 25 °C: conventional extraction at 25 °C; CONV 60 °C conventional extraction at 60 °C; HPUS: high power ultrasounds; US ultrasounds; HHP: high hydrostatic pressure; GA: Gallic Acid; *p*OH-B: *p*-Hydroxybenzoic Acid; SyrA: Syringic Acid; EllA: Ellagic Acid; ProCA: Protocatechuic Acid; Cat; Catechin; EGC: Epigallocatechin; ChlA: Chlorogenic Acid; ChicA Chicoric Acid; CafA: Caffeic Acid; FerA: Ferulic Acid; pCuA: *p*-Cumaric Acid; Rut: Rutin; XAN: Xanthohumol; TOT: Total; n.d.: not detected. Data on columns with different letters were statistically different at *p*-level < 0.05.

**Table 2:** Functional properties of different food grade hop extracts.

Extraction Method	Temperature (°C)	Time (min)	TPC	sd	TFC	sd	TEAC	sd	IC_50_	sd	Chl *α*	sd	Chl *β*	sd	TCC	sd
			(mg GAE g^−1^ dm)		(mg QE g^−1^ dm)		(µmol g^−1^ dm)		(mg mL^−1^)		(mg g^−1^dm)					
CONV	25	15	35.8 ^gh^	0.11	18.4 ^def^	0.02	173 ^il^	4	0.070 ^b^	0.004	0.369 ^l^	0.017	0.307 ^f^	0.067	1.14 ^h^	0.01
		30	34.8 ^gh^	0.11	19.3 ^d^	0.03	197 ^gh^	6	0.056 ^c^	0.001	0.491 ^hi^	0.005	0.448 ^ef^	0.042	1.26 ^gh^	0.04
		60	34.2 ^h^	1.56	18.9 ^de^	0.02	232 ^cd^	2	0.056 ^c^	0.001	0.468 ^il^	0.012	0.427 ^ef^	0.024	1.25 ^gh^	0.01
		120	35.4 ^gh^	0.12	21.1 ^c^	0.13	230 ^cde^	2	0.057 ^c^	0.002	0.609 ^fg^	0.002	0.486 ^ef^	0.030	1.32 ^g^	0.02
CONV	60	15	41.2 ^f^	1.56	28.2 ^a^	0.02	224 ^def^	0	0.050 ^cde^	0.001	0.981 ^c^	0.051	1.043 ^c^	0.084	1.84 ^cd^	0.04
		30	41.7 ^f^	0.42	27.1 ^a^	0.15	182 ^hi^	1	0.049 ^de^	0.004	1.34 ^ab^	0.01	1.29 ^b^	0.04	1.95 ^bc^	0.02
		60	44.3 ^cd^	0.63	27.6 ^a^	0.04	161 ^l^	3	0.053 ^cd^	0.001	0.758 ^de^	0.038	0.887 ^c^	0.092	1.82 ^d^	0.21
		120	45.3 ^bc^	0.09	23.0 ^b^	0.16	245 ^bc^	1	0.044 ^ef^	0.001	1.17 ^ab^	0.05	1.46 ^ab^	0.05	2.06 ^ab^	0.01
HPUS	25	15	36.6 ^g^	1.72	19.1 ^d^	1.51	187 ^hi^	3	0.035 ^g^	0.004	0.590 ^gh^	0.074	0.589 ^e^	0.009	1.52 ^f^	0.12
		30	35.8 ^gh^	1.96	17.2 ^fgh^	0.17	221 ^def^	14	0.039 ^fg^	0.004	0.713 ^ef^	0.084	0.835 ^cd^	0.078	1.59 ^ef^	0.02
		60	41.2 ^f^	0.20	18.2 ^def^	0.21	239 ^c^	16	0.037 ^g^	0.004	0.852 ^d^	0.013	1.03 ^c^	0.01	1.74 ^de^	0.01
		120	42.1 ^ef^	1.46	19.0 ^d^	1.30	205 ^g^	13	0.032 ^g^	0.001	0.853 ^d^	0.062	0.926 ^c^	0.014	1.76 ^d^	0.02
US	25	15	43.4 ^de^	1.37	16.0 ^i^	0.41	214 ^efg^	6	0.054 ^cd^	0.001	0.848 ^d^	0.052	1.43 ^ab^	0.05	1.73 ^de^	0.04
		30	45.0 ^cd^	0.60	16.2 ^hi^	0.28	271 ^a^	3	0.052 ^cd^	0.002	1.19 ^a^	0.04	1.61 ^a^	0.04	2.20 ^a^	0.07
		60	46.9 ^b^	1.80	17.7 ^efg^	0.55	258 ^ab^	8	0.039 ^fg^	0.002	0.998 ^c^	0.011	1.54 ^ab^	0.01	2.01 ^bc^	0.12
		120	48.5 ^a^	2.94	16.9 ^ghi^	0.26	259 ^ab^	11	0.049 ^de^	0.011	1.08 ^bc^	0.14	1.51 ^ab^	0.12	2.06 ^ab^	0.07
HHP	25	5	36.8 ^g^	0.15	13.1 ^l^	1.10	209 ^fg^	1	0.081 ^a^	0.004	0.384 ^il^	0.038	0.604 ^de^	0.056	1.30 ^g^	0.05

CONV: conventional; HPUS: High Power Ultrasounds; US ultrasounds; HHP: High Hydrostatic Pressure; sd: standard deviation; TPC: Total Phenolic Content (mg GAE g^−1^ dm); TFC Total Flavonoids Content (mg QE g^−1^ dm); TEAC: Trolox Equivalent Antioxidant Capacity (µmol g^−1^ dm); antiradical capacity express as Inhibitory Capacity IC_50_ (mg mL^−1^); Chl α: Chlorophyll α; Chl *β*: Chlorophyll *β* and TCC: Total Carotenoid Content (mg^−1^). Data on columns with different letters were statistically different at *p*-level < 0.05.

## Supplementary Materials

The following are available on line at https://www.mdpi.com/article/10.3390/plants11010041/s1. Table S1: Multifactorial ANOVA of the individual and interactive effects of extraction temperature (T) and time (t) for both conventional extractions (25 °C; 60 °C), and extraction method (EM) and time (t) for both ultrasound assisted extractions (US; HPUS), on the functional properties of hop extracts, Table S2: Multifactorial ANOVA of the individual and interactive effects of extraction temperature (T) and time (t) for both conventional extractions (25 °C; 60 °C), and extraction method (EM) and time (t) for both ultrasound assisted extractions (US; HPUS), on the functional properties of hop extracts, Table S3: Predictions values on variable t.
