# Supplementary material for: Use of Conventional and Innovative Technologies for the Production of Food Grade Hop Extracts: Focus on Bioactive Compounds and Antioxidant Activity
Source: Plants (Basel). 2021 Dec 23;11(1):41. doi: 10.3390/plants11010041 (PMC8747399; doi:10.3390/plants11010041)
Supplement: Supplementary file 1 [file plants-11-00041-s001.zip › Supplementary material/TABLE_S1.pdf]

Table S1. Multifactorial ANOVA of the individual and interactive effects of extraction temperature (T) and time (t) for both conventional extractions (25°C; 60°C), and extraction method (EM) and time (t) for both ultrasound assisted extractions (US; HPUS), on the functional properties of hop extracts.

			GA	pOH-B	SyrA	EllA	ProCA	Cat	EGC	ChlA	ChicA	CafA	FerA	pCuA	Rut	XAN	TOT
F	CONV	T	n.d.	100.91***	209.38***	51.374***	23.366**	116.64***	197.701***	1776.1***	n.d.	197.263***	39.196***	408.235***	107.08***	142.374***	495.33***
F		t	n.d.	1575.49***	27.01***	91.474***	n.s.	529.23***	6.747*	806.4***	n.d.	75.291***	10.7641**	33.779***	82.40***	n.s.	17.82***
F		T x t	n.d.	12.5**	n.s.	n.s.	24.375***	n.s.	16.517***	657.5***	n.d.	13.5148**	6.137*	23.1756***	7.78**	8.345**	n.s.
F	UAE	EM	49.94***	94.95***	26.727***	40.39***	n.s.	n.s.	21.38**	9.15*	74.298***	n.s.	12.167**	76.395***	6.62*	44.658***	n.s.
F		t	2868.68***	63.88***	10.043**	391.50***	n.s.	398.18***	109.73***	43.78***	22.357***	43.531***	29.850***	71.341***	23.97***	10.087**	66.47***
F		EM x t	9.54**	5.25*	7.083*	11.31**	n.s.	7.28*	9.75**	28.88***	22.357***	6.769*	n.s.	11.851**	17.73***	n.s.	4.31*

CONV: conventional extraction; UAE; ultrasound assisted extraction; GA: Gallic Acid; pOH-B: p-Hydroxybenzoic Acid; SyrA: Syringic Acid; EllA: Ellagic Acid; ProCA: Protocatechuic Acid; Cat; Catechin; EGC: Epigallocatechin; ChlA: Chlorogenic Acid; ChicA Chicoric Acid; CafA: Caffeic Acid; FerA: Ferulic Acid; pCuA: p-Coumaric Acid; Rut: Rutin; XAN: Xanthohumol; TOT: Total; n.d. not detected; n.s. not significant; *$p < 0.05$; **$p < 0.01$; ***$p < 0.001$.
