# Supplementary material for: Use of Conventional and Innovative Technologies for the Production of Food Grade Hop Extracts: Focus on Bioactive Compounds and Antioxidant Activity
Source: Plants (Basel). 2021 Dec 23;11(1):41. doi: 10.3390/plants11010041 (PMC8747399; doi:10.3390/plants11010041)
Supplement: Supplementary file 1 [file plants-11-00041-s001.zip › Supplementary material/TABLE_S2.pdf]

Table S2. Multifactorial ANOVA of the individual and interactive effects of extraction temperature (T) and time (t) for both conventional extractions (25°C; 60°C), and extraction method (EM) and time (t) for both ultrasound assisted extractions (US; HPUS), on the functional properties of hop extracts.

			TPC	TFC	TEAC	IC_{50}	Chl α	Chl β	TCC
F		T	727***	22835.1***	10.57*	1348.7***	1244.48***	656.265***	334.393**
F	CONV	t	10.0***	170.4***	217.75***	223.2***	71.92***	27.023***	6.455*
F		T x t	15.3***	1458.1***	300.51***	157.6***	29.35**	13.678**	n.s.
F		EM	184.96***	39.92***	64.691***	30.972**	60.367***	76.2506***	80.233***
F	UAE	t	26.94***	5.25**	19.894***	n.s.	10.562**	n.s.	12.335**
F		EM x t	n.s.	4.73**	3.404*	n.s.	4.410*	n.s.	5.694*

CONV: conventional extraction; UAE Ultrasound Assisted Extraction; n.s.: not significant; TPC: Total Polyphenol Content (mg GAE g^{-1} dm); TFC: Total Flavonoids Content (mg QE g^{-1} dm); TEAC: Trolox Equivalent Antioxidant Capacity (μmol g^{-1} dm); antiradical capacity express as Inhibitory Capacity IC_{50} (mg/ml); Chl α: Chlorophyll α; Chl β: Chlorophyll β and TCC: Total Carotenoid Content (mg g dm^{-1}). **p* < 0,05; ***p* < 0,01; ****p* < 0,001.
