# Supplementary material for: Use of Conventional and Innovative Technologies for the Production of Food Grade Hop Extracts: Focus on Bioactive Compounds and Antioxidant Activity
Source: Plants (Basel). 2021 Dec 23;11(1):41. doi: 10.3390/plants11010041 (PMC8747399; doi:10.3390/plants11010041)
Supplement: Supplementary file 1 [file plants-11-00041-s001.zip › Supplementary material/TABLE_S3.pdf]

Table S3. Predictions values on variable t:

Observation	t	Pred(t)	Residual
HPUS 15'	15,0	18,3	-3,336
HPUS 15'	15,0	13,6	1,383
HPUS 30'	30,0	19,3	10,714
HPUS 30'	30,0	11,2	18,848
HPUS 60'	60,0	65,7	-5,717
HPUS 60'	60,0	84,7	-24,703
HPUS 120'	120,0	98,6	21,411
HPUS 120'	120,0	107,2	12,796
US 15'	15,0	5,3	9,740
US 15'	15,0	10,1	4,863
US 30'	30,0	26,7	3,325
US 30'	30,0	33,9	-3,895
US 60'	60,0	74,0	-14,038
US 60'	60,0	75,3	-15,256
US 120'	120,0	105,6	14,402
US 120'	120,0	104,1	15,941
CONV 60°C 15'	15,0	16,6	-1,597
CONV 60°C 15'	15,0	12,3	2,716
CONV 60°C 30'	30,0	36,6	-6,604
CONV 60°C 30'	30,0	38,9	-8,869
CONV 60°C 60'	60,0	98,1	-38,058
CONV 60°C 60'	60,0	101,7	-41,743
CONV 60°C 120'	120,0	105,5	14,539
CONV 60°C 120'	120,0	111,7	8,322
CONV 25°C 15'	15,0	12,5	2,526
CONV 25°C 15'	15,0	10,0	5,020
CONV 25°C 30'	30,0	28,9	1,120
CONV 25°C 30'	30,0	35,1	-5,051
CONV 25°C 60	60,0	67,6	-7,641
CONV 25°C 60	60,0	78,6	-18,638
CONV 25°C 120'	120,0	77,2	42,793
CONV 25°C 120'	120,0	80,7	39,338
HHP	5,0	21,9	-16,904
HHP	5,0	22,7	-17,748
